# Trends in the Prevalence of Chronic Obstructive Pulmonary Disease Among Adults Aged ≥18 Years — United States, 2011–2021

**DOI:** 10.15585/mmwr.mm7246a1

**Published:** 2023-11-17

**Authors:** Yong Liu, Susan A. Carlson, Kathleen B. Watson, Fang Xu, Kurt J. Greenlund

**Affiliations:** ^1^Division of Population Health, National Center for Chronic Disease Prevention and Health Promotion, CDC; ^2^Division of Diabetes Translation, National Center for Chronic Disease Prevention and Health Promotion, CDC.

**Summary**

**What is already known about this topic?**

Demographic disparities in chronic obstructive pulmonary disease (COPD) prevalence have been reported. COPD prevalence among adults aged ≥25 years declined during 1999–2011.

**What is added by this report?**

From 2011 to 2021, prevalence of COPD among adults remained stable overall (6.1% to 6.0%) and in most subgroups and states; prevalence increased among adults aged ≥75 years, those living in rural areas, and those who ever smoked. Disparities based on rural residence and smoking status increased.

**What are the implications for public health practice?**

Evidence-based strategies, especially those tailored for groups disproportionately affected, can reduce COPD prevalence and address the continued need for prevention, early diagnosis, treatment, and management.

## Abstract

Chronic obstructive pulmonary disease (COPD) is a leading cause of death in the United States. Overall COPD prevalence declined during 1999–2011. Trends in COPD prevalence during the previous decade have not been reported. CDC analyzed 2011–2021 Behavioral Risk Factor Surveillance System data to assess trends and differences in self-reported physician-diagnosed COPD prevalence among U.S. adults aged ≥18 years. Age-standardized prevalence of COPD did not change significantly from 2011 (6.1%) to 2021 (6.0%). Prevalence was stable for most states and subgroups; however, it decreased significantly among adults aged 18–44 years (average annual percent change [AAPC] = −2.0%) and increased significantly among those aged ≥75 years (AAPC = 1.3%), those living in micropolitan counties (0.8%), and among current (1.5%) or former (1.2%) smokers. COPD prevalence remained elevated in the following groups: women, adults aged ≥65 years, those with a lower education level, unable to work, living in rural areas, and who ever smoked. Evidence-based strategies, especially those tailored for adults disproportionately affected, can reduce COPD prevalence, and address the continued need for prevention, early diagnosis, treatment, and management.

## Introduction

Chronic obstructive pulmonary disease (COPD) is a group of progressive lung diseases, including emphysema and chronic bronchitis. COPD accounts for most of the deaths from chronic lower respiratory diseases, the sixth leading cause of death in the United States in 2021 (*1*). Elevated prevalence of COPD has been reported in the following groups: women, older adults (aged ≥65 years), residents in rural areas, adults with a lower education level, and those who ever smoked (*2*). During 1999–2011, estimates from the National Health Interview Survey (NHIS) indicated that the prevalence of self-reported physician-diagnosed COPD significantly declined among U.S. adults (aged ≥25 years) overall and among adults aged 25–44 years (*3*). Trends and differences in COPD prevalence during the previous decade have not been reported overall and by subgroups.

## Methods

### Data Collection 

The Behavioral Risk Factor Surveillance System (BRFSS) is an annual state-based, random-digit–dialed mobile and landline telephone survey among noninstitutionalized U.S. adults aged ≥18 years; the survey covers all 50 states, the District of Columbia (DC), and U.S. territories.* The median survey response rate for all states and DC was 49.7% in 2011^†^ and 43.8% in 2021.^§^ The analytic sample included respondents with complete data for COPD, sex, age, race and ethnicity, education, employment, urban-rural status, and smoking status (2011: 478,788 [96.2% of respondents had complete information]; 2021^¶^: 386,439 [89.5% of respondents had complete information]). Self-reported physician-diagnosed COPD was defined as a “yes” response to the question, “Has a doctor, nurse, or other health professional ever told you that you had chronic obstructive pulmonary disease or COPD, emphysema, or chronic bronchitis?” 

### Data Analysis

CDC estimated age-specific or age-standardized prevalence (standardized to the 2000 projected U.S. population)** of COPD overall, by selected characteristics including urban-rural status,^††^ and by state. Overall and for all subgroups, linear and nonlinear trends in COPD prevalence during 2011–2021 were assessed using permutation tests in Joinpoint trend analysis software (version 4.8.0.1; National Cancer Institute^§§^). Annual percent change (APC) for each line segment (when joinpoints were identified) and average annual percent change (AAPC) from 2011 to 2021 were estimated. Differences by selected characteristics (compared with a reference category) in COPD prevalence for years 2011 and 2021 were assessed using *t*-tests. Linear trend tests were performed using orthogonal polynomial contrasts for ordinal variables.^¶¶^ The statistical significance level for all the tests was set at alpha = 0.05. Analyses were conducted using SAS software (version 9.4; SAS Institute) and SAS-callable SUDAAN software (version 11.0.1; RTI International) to account for the complex sample design and weighting. This activity was reviewed by CDC, deemed not research, and was conducted consistent with applicable federal law and CDC policy.***

## Results

### Differences by Sociodemographic Characteristics

An estimated 6.4% of U.S. adults (population estimate = 14.3 million) in 2011 and 6.5% (14.2 million) in 2021 had COPD (Table 1). In 2011 and 2021, age-standardized COPD prevalence was higher among women than among men, higher among non-Hispanic American Indian or Alaska Native and non-Hispanic other persons than among non-Hispanic White persons, higher among persons who were unemployed, retired, homemakers or students, and unable to work than among those who were employed, and higher among adults who were current or former smokers than among never smokers; prevalence was lower among non-Hispanic Asian, Native Hawaiian, Pacific Islander, or Hispanic persons than among non-Hispanic White persons. COPD prevalence increased with increasing age, decreasing education level, and decreasing urbanicity.

**TABLE 1:** Trends and differences in prevalence of chronic obstructive pulmonary disease among adults aged ≥18 years, by sociodemographic characteristics — Behavioral Risk Factor Surveillance System, United States, 2011–2021

Characteristic	2011*			2021*			2011–2021		
	Sample size	No. of adults with COPD (x1,000)	% (95% CI)	Sample size	No. of adults with COPD (x1,000)	% (95% CI)	AAPC, % (95% CI)	No. of joinpoints^†^	Segment-specific APC, % (95% CI)
**Overall**									
Crude	478,788	14,276	6.4 (6.2 to 6.5)	386,439	14,170	6.5 (6.4 to 6.7)	0.4 (–0 to 0.9)	0	—^§^
Age-standardized^¶^	478,788	14,276	6.1 (6.0 to 6.3)	386,439	14,170	6.0 (5.9 to 6.2)	0.0 (–0.6 to 0.6)	0	—
**Sex^¶^**									
Men (Ref)	187,876	5,877	5.4 (5.2 to 5.5)	178,716	6,154	5.5 (5.3 to 5.7)	0.2 (–0.3 to 0.6)	0	—
Women	290,912	8,399	6.9 (6.7 to 7.0)**	207,723	8,016	6.5 (6.3 to 6.7)**	–0.3 (–0.8 to 0.2)	0	—
**Age group, yrs^††^**									
18–44	130,837	3,443	3.2 (3.0 to 3.4)	117,294	2,739	2.7 (2.6 to 2.9)	–2.0 (–3.1 to –0.9)^§§^	0	—
45–64	195,611	6,044	7.8 (7.6 to 8.1)	130,157	5,368	7.9 (7.6 to 8.2)	–0.1 (–1.3 to 1.1)	1	2011–2018: 1.1 (0.1 to 2.1)^§§^ 2018–2021: –2.8 (–6.9 to 1.4)
65–74	82,898	2,634	12.3 (11.8 to 12.7)	80,941	3,462	12.1 (11.6 to 12.7)	0.4 (–0.3 to 1.0)	0	—
≥75	69,442	2,156	11.8 (11.4 to 12.3)	58,047	2,600	13.2 (12.5 to 13.9)	1.3 (0.2 to 2.3)^§§^	0	—
**Race and ethnicity^¶^**									
Hispanic or Latino	30,662	1,071	4.1 (3.7 to 4.5)**	30,697	1,261	3.9 (3.5 to 4.4)**	–0.3 (–2.1 to 1.6)	0	—
American Indian or Alaska Native, non-Hispanic	6,794	256	10.4 (9.0 to 11.9)**	6,555	225	10.2 (8.8 to 11.8)**	0.1 (–1.2 to 1.5)	0	—
Asian, Native Hawaiian, or Pacific Islander, non-Hispanic	9,328	179	2.3 (1.7 to 2.9)**	10,743	209	1.9 (1.2 to 2.8)**	0.6 (–2.4 to 3.6)	0	—
Black or African-American, non-Hispanic	39,277	1,546	6.2 (5.8 to 6.7)	28,213	1,633	6.2 (5.7 to 6.7)	–0.7 (–2.0 to 0.6)	0	—
White, non-Hispanic (Ref)	381,484	10,799	6.4 (6.3 to 6.6)	298,583	10,503	6.5 (6.3 to 6.7)	0.2 (–0.3 to 0.6)	0	—
Other, non-Hispanic	11,243	426	10.7 (9.4 to 12.0)**	11,648	339	8.0 (7.1 to 9.1)**	–2.1 (–3.3 to –0.9)^§§^	0	—
**Education^¶,††^**									
Less than high school diploma	42,171	3,511	9.9 (9.4 to 10.4)	22,115	2,921	10.4 (9.7 to 11.1)	0.2 (–0.8 to 1.3)	0	—
High school diploma or GED	142,038	4,946	7.1 (6.8 to 7.4)	97,878	4,513	7.3 (7.0 to 7.6)	0.6 (–0.1 to 1.4)	0	—
Some college or technical school	129,392	4,132	6.2 (6.0 to 6.5)	107,182	4,774	6.6 (6.4 to 6.9)	0.6 (0.2 to 0.9)^§§^	0	—
College graduate	165,187	1,686	2.9 (2.8 to 3.1)	159,264	1,961	2.7 (2.5 to 2.8)	–0.7 (–1.5 to 0.1)	0	—
**Employment status^¶^**									
Employed (Ref)	237,171	3,978	3.7 (3.5 to 3.9)	200,549	4,032	3.7 (3.5 to 3.9)	–0.2 (–1.0 to 0.6)	0	—
Unemployed	29,270	1,469	8.1 (7.5 to 8.7)**	18,631	976	7.7 (7.0 to 8.6)**	–0.5 (–2.0 to 1.0)	0	—
Retired	134,809	4,157	8.5 (6.1 to 11.6)**	119,126	5,181	11.0 (7.6 to 15.6)**	1.2 (–6.0 to 8.9)	1	2011–2017: –5.9 (–13.8 to 2.7) 2017–2021: 12.8 (–6.6 to 36.2)
Unable to work	34,197	3,556	20.8 (19.8 to 21.8)**	22,876	3,186	19.3 (18.2 to 20.4)**	–0.9 (–1.3 to –0.5)^§§^	0	—
Homemaker or student	43,341	1,115	5.1 (4.8 to 5.5)**	25,257	795	5.6 (4.8 to 6.4)**	0.7 (–0.8 to 2.2)	0	—
**Urban-rural status^¶††^**									
Large central metropolitan	75,505	3,330	5.2 (4.9 to 5.5)	57,337	3,266	4.8 (4.5 to 5.2)	–0.7 (–1.6 to 0.2)	0	—
Large fringe metropolitan	86,425	3,100	5.6 (5.3 to 5.9)	74,496	3,238	5.4 (5.1 to 5.7)	–0.2 (–1.1 to 0.6)	0	—
Medium metropolitan	106,501	3,117	6.3 (6.1 to 6.6)	80,224	3,033	6.5 (6.1 to 6.8)	0.2 (–0.2 to 0.7)	0	—
Small metropolitan	63,723	1,540	6.9 (6.5 to 7.3)	54,798	1,493	6.7 (6.3 to 7.1)	–0.4 (–1.2 to 0.4)	0	—
Micropolitan	73,761	1,734	7.6 (7.2 to 8.0)	62,619	1,738	8.0 (7.5 to 8.4)	0.8 (0.2 to 1.4)^§§^	0	—
Noncore	72,873	1,452	7.8 (7.4 to 8.3)	56,965	1,401	8.2 (7.7 to 8.8)	0.4 (–0.7 to 1.5)	1	2011–2018: 1.7 (0.8 to 2.7)^§§^ 2018–2021: –2.7 (–6.4 to 1.2)
**Smoking status^¶^**									
Current smoker	80,833	5,585	13.7 (13.3 to 14.2)**	50,637	4,943	16.2 (15.6 to 16.9)**	1.5 (1.1 to 1.8)^§§^	0	—
Former smoker	141,395	5,219	7.0 (6.7 to 7.4)**	106,928	5,453	7.7 (7.3 to 8.0)**	1.2 (0.5 to 2.0)^§§^	0	—
Never smoker (Ref)	256,560	3,473	2.9 (2.7 to 3.0)	228,874	3,774	2.8 (2.6 to 2.9)	–0.4 (–1.2 to 0.4)	0	—

**Abbreviations**: AAPC = average annual percent change; APC = annual percent change; COPD = chronic obstructive pulmonary disease; GED = general educational development certificate; Ref = referent group.

* Estimates were calculated using sampling weights. The analytic sample included respondents with complete data for COPD, sex, age, race or ethnicity, education, employment status, urban-rural status, and smoking status (weighted estimate for 2011: 224.4 million [95.4% of weighted sample had complete information]; 2021: 216.5 million [89.0% of weighted sample had complete information]). Florida was unable to collect data during enough months to meet the minimum requirements for inclusion in the 2021 public-use dataset.

^†^ Indicates a nonlinear trend if the number of joinpoints is equal to one or more.

^§^ Dashes indicate that no joinpoints (no line segments) were identified using a permutation test in the best-fit joinpoint model.

^¶^ Age-standardized COPD prevalence was calculated using the 2000 U.S. Census Bureau projected U.S. adult population with five age groups (18–24, 25–34, 35–44, 45–64, and ≥65 years) Distribution #9. https://www.cdc.gov/nchs/data/statnt/statnt20.pdf

** Indicates statistically significant difference on the basis of *t*-tests in the COPD prevalence between the reported level of each characteristic and the Ref (p<0.05).

^††^ Indicates significant linear trend across categories within each (2011 and 2021) year (p<0.05).

^§§^ Indicates significant linear trend across years using a permutation test (p<0.05).

### Trends Over Time

Age-standardized prevalence of COPD from 2011 to 2021 remained stable overall (6.1% in 2011 to 6.0% in 2021; AAPC = 0%) and for most subgroups (Table 1). Significant increases occurred among adults aged ≥75 years (AAPC = 1.3%), respondents with some college or technical school education (AAPC = 0.6%), those living in micropolitan counties (AAPC = 0.8%), and adults who were current smokers (AAPC = 1.5%) or former smokers (AAPC = 1.2%) (Table 1) (Figure). COPD prevalence increased significantly from 2011 to 2018 and remained stable from 2018 to 2021 among adults aged 45–64 years and those living in noncore areas (Table 1). COPD prevalence decreased among adults aged 18–44 years (AAPC = −2.0%) and those who were unable to work (AAPC = −0.9%). Age-standardized COPD prevalence in 2011 ranged from 3.9% in Minnesota to 9.5% in Kentucky and in 2021 from 3.0% in Hawaii to 11.8% in West Virginia (Table 2). From 2011 to 2021, age-standardized COPD prevalence increased significantly in Louisiana (AAPC = 2.4%) and decreased significantly in Hawaii (AAPC = −2.5%), New Mexico (AAPC = −2.4%), Maryland (AAPC = −2.0%), Massachusetts (AAPC = −2.0%), and New York (AAPC = −1.6%). Statistically significant increases in COPD prevalence occurred in Colorado from 2014 to 2021, Utah from 2015 to 2021, and West Virginia from 2011 to 2017; decreases occurred from 2013 to 2021 in Arizona, DC, Washington, and Wyoming.

**FIGURE:**
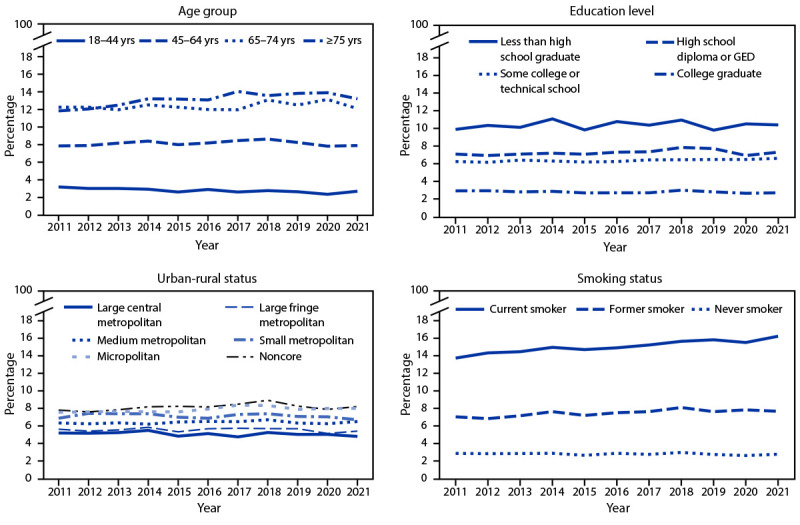
Prevalence* of chronic obstructive pulmonary disease among adults aged ≥18 years, by selected characteristics — Behavioral Risk Factor Surveillance System, United States, 2011–2021 **Abbreviation: **GED = general educational development certificate. *Estimates were calculated using sampling weights and estimates by education level, urban-rural status, and smoking status were age-standardized using the 2000 Census Bureau projected U.S. adult population with five age groups (18–24, 25–34, 35–44, 45–64, and ≥65 years) Distribution #9. https://www.cdc.gov/nchs/data/statnt/statnt20.pdf

**TABLE 2:** Trends in prevalence* of chronic obstructive pulmonary disease among adults aged ≥18 years, by jurisdiction — Behavioral Risk Factor Surveillance System, United States, 2011–2021

Jurisdiction	2011	2021	2011–2021		
	% (95% CI)	% (95% CI)	AAPC % (95% CI)	No. of joinpoints^†^	Segment-specific APC, % (95% CI)
Alabama	9.3 (8.4 to 10.2)	8.6 (7.5 to 9.8)	–0.4 (–1.5 to 0.7)	0	—^§^
Alaska	5.9 (4.9 to 7.1)	5.5 (4.7 to 6.3)	–0.7 (–3.3 to 2.0)	0	—
Arizona	5.1 (4.4 to 5.9)	5.0 (4.4 to 5.6)	0.5 (–4.2 to 5.5)	1	2011–2013: 15.1 (–13.8 to 53.6) 2013–2021: –2.8 (–5.1 to –0.5)^¶^
Arkansas	7.4 (6.5 to 8.4)	8.9 (7.8 to 9.9)	1.3 (–0.1 to 2.8)	0	—
California	4.5 (4.1 to 4.9)	4.4 (3.8 to 5.1)	–0.3 (–1.4 to 0.9)	0	—
Colorado	4.7 (4.2 to 5.2)	4.9 (4.4 to 5.4)	–0.5 (–2.3 to 1.3)	1	2011–2014: –6.0 (–12.0 to 0.2) 2014–2021: 1.9 (0.1 to 3.8)^¶^
Connecticut	5.8 (4.9 to 6.8)	4.6 (4.0 to 5.2)	–1.2 (–2.8 to 0.3)	0	—
Delaware	4.9 (4.2 to 5.7)	5.7 (4.7 to 6.6)	0.9 (–1.7 to 3.5)	0	—
District of Columbia	4.8 (4.0 to 5.7)	4.8 (3.9 to 5.8)	0.4 (–2.6 to 3.5)	1	2011–2013: 15.2 (–3.3 to 37.1) 2013–2021: –3.0 (–4.9 to –1.1)^¶^
Florida	7.3 (6.6 to 8.1)	— **	–0.2 (–2.1 to 1.7)**	0	—
Georgia	7.0 (6.4 to 7.8)	6.2 (5.4 to 7.0)	–1.2 (–4.2 to 1.9)	1	2011–2019: 0.6 (–1.0 to 2.3) 2019–2021: –8.2 (–23.0 to 9.7)
Hawaii	4.2 (3.6 to 4.9)	3.0 (2.5 to 3.5)	–2.5 (–4.4 to –0.5)^¶^	0	—
Idaho	5.1 (4.4 to 5.9)	5.3 (4.6 to 5.9)	0.8 (–0.6 to 2.2)	0	—
Illinois	6.0 (5.2 to 7.0)	5.0 (4.1 to 5.9)	–0.1 (–2.0 to 1.8)	0	—
Indiana	8.0 (7.3 to 8.7)	7.8 (7.1 to 8.4)	0.5 (–0.6 to 1.7)	0	—
Iowa	4.7 (4.2 to 5.4)	6.0 (5.3 to 6.6)	0.9 (–0.7 to 2.5)	0	—
Kansas	6.3 (5.9 to 6.8)	5.8 (5.4 to 6.2)	–0.3 (–1.2 to 0.6)	0	—
Kentucky	9.5 (8.7 to 10.5)	10.2 (9.2 to 11.3)	0.3 (–1.2 to 1.9)	0	—
Louisiana	6.6 (6.0 to 7.4)	8.2 (7.2 to 9.2)	2.4 (1.1 to 3.8)^¶^	0	—
Maine	7.0 (6.5 to 7.6)	7.4 (6.7 to 8.1)	0.7 (–0.7 to 2.1)	0	—
Maryland	5.8 (5.1 to 6.6)	4.4 (4.0 to 4.9)	–2.0 (–3.2 to –0.7)^¶^	0	—
Massachusetts	5.5 (5.1 to 6.0)	5.4 (4.6 to 6.1)	–2.0 (–3.8 to –0.1)^¶^	0	—
Michigan	7.5 (6.8 to 8.3)	7.4 (6.7 to 8.1)	0.0 (–1.5 to 1.4)	0	—
Minnesota	3.9 (3.5 to 4.4)	4.2 (3.8 to 4.6)	0.3 (–0.8 to 1.5)	0	—
Mississippi	8.1 (7.4 to 9.0)	8.7 (7.6 to 9.8)	1.6 (–0.4 to 3.6)	0	—
Missouri	7.7 (6.9 to 8.7)	7.7 (7.0 to 8.4)	0.5 (–0.6 to 1.5)	0	—
Montana	5.5 (4.9 to 6.3)	4.9 (4.3 to 5.6)	–0.6 (–2.5 to 1.3)	0	—
Nebraska	4.8 (4.4 to 5.1)	5.2 (4.7 to 5.7)	0.7 (–0.5 to 2.0)	0	—
Nevada	7.2 (6.2 to 8.4)	6.0 (4.9 to 7.0)	–1.0 (–2.2 to 0.2)	0	—
New Hampshire	6.0 (5.3 to 6.8)	6.4 (5.5 to 7.3)	0.3 (–1.7 to 2.3)	0	—
New Jersey	5.0 (4.5 to 5.5)	4.9 (4.3 to 5.6)	1.1 (–2.3 to 4.7)^††^	0	—
New Mexico	5.9 (5.4 to 6.6)	4.9 (4.2 to 5.5)	–2.4 (–3.7 to –1.1)^¶^	0	—
New York	5.8 (5.1 to 6.5)	5.0 (4.6 to 5.4)	–1.6 (–2.9 to –0.3)^¶^	0	—
North Carolina	6.6 (6.0 to 7.3)	7.1 (6.1 to 8.1)	0.2 (–1.2 to 1.7)	0	—
North Dakota	4.6 (4.0 to 5.4)	4.5 (3.8 to 5.2)	1.5 (–0.3 to 3.2)	0	—
Ohio	7.2 (6.5 to 7.9)	7.9 (7.2 to 8.6)	0.3 (–0.6 to 1.2)	0	—
Oklahoma	8.2 (7.4 to 8.9)	7.4 (6.5 to 8.2)	0.5 (–0.7 to 1.7)	0	—
Oregon	5.5 (4.9 to 6.3)	5.4 (4.7 to 6.1)	–0.6 (–2.3 to 1.2)	0	—
Pennsylvania	6.2 (5.6 to 6.9)	6.2 (5.4 to 6.9)	0.2 (–0.7 to 1.0)	0	—
Rhode Island	5.9 (5.2 to 6.7)	5.2 (4.4 to 6.0)	–0.4 (–2.5 to 1.8)	0	—
South Carolina	7.1 (6.5 to 7.7)	6.9 (6.1 to 7.6)	0.2 (–0.7 to 1.1)	0	—
South Dakota	5.1 (4.3 to 6.0)	5.3 (3.9 to 6.6)	0.4 (–2.3 to 3.2)	0	—
Tennessee	8.8 (7.3 to 10.5)	9.5 (8.4 to 10.5)	0.3 (–1.1 to 1.7)	0	—
Texas	5.6 (5.1 to 6.2)	6.0 (5.2 to 6.8)	0.0 (–1.4 to 1.5)	0	—
Utah	4.3 (3.9 to 4.8)	4.5 (4.0 to 4.9)	0.3 (–1.2 to 1.9)	1	2011–2015: –2.5 (–6.0 to 1.1) 2015–2021: 2.2 (0.0 to 4.4)^¶^
Vermont	4.5 (4.0 to 5.2)	5.6 (4.8 to 6.4)	0.9 (–0.6 to 2.5)	0	—
Virginia	6.0 (5.3 to 6.8)	6.2 (5.5 to 6.8)	–0.1 (–1.4 to 1.3)	0	—
Washington	4.0 (3.6 to 4.6)	4.8 (4.3 to 5.2)	0.5 (–2.9 to 4.0)	1	2011–2013: 15.4 (–5.4 to 40.7) 2013–2021: –2.9 (–4.8 to –0.9)^¶^
West Virginia	8.3 (7.4 to 9.2)	11.8 (10.8 to 12.7)	2.5 (–1.3 to 6.3)	1	2011–2017: 7.8 (2.6 to 13.3)^¶^ 2017–2021: –5.1 (–13.0 to 3.7)
Wisconsin	5.1 (4.2 to 6.2)	5.0 (4.1 to 6.0)	–0.2 (–1.9 to 1.4)	0	—
Wyoming	6.0 (5.3 to 6.8)	5.6 (4.7 to 6.5)	–1.0 (–2.9 to 0.9)	1	2011–2013: 6.6 (–4.4 to 18.8) 2013–2021: –2.9 (–4.1 to –1.6)^¶^

**Abbreviations:** AAPC = average annual percent change; APC = annual percent change; COPD = chronic obstructive pulmonary disease.

* Estimates were calculated using sampling weights and age-standardized using the 2000 U.S. Census Bureau projected U.S. adult population with five age groups (18–24, 25–34, 35–44, 45–64, and ≥65 years) Distribution #9. (https://www.cdc.gov/nchs/data/statnt/statnt20.pdf). The analytic sample included respondents with complete data for COPD, sex, age, race and ethnicity, education, employment status, urban-rural status, and smoking status (2011: 478,788 respondents; 2021: 386,439).

^†^ Indicates a nonlinear trend if the number of joinpoints is equal to one or more.

^§^ Dashes indicate that no joinpoints (no line segments) were identified using a permutation test in the best-fit joinpoint model.

^¶^ Significant linear trend across years using a permutation test (p<0.05).

** Respondents in Florida were not included in 2021. AAPC was derived on the basis of data available during 2011–2020 (COPD prevalence = 6.2 [5.4–7.1] in 2020).

^††^ Respondents in New Jersey were not included in 2019. AAPC was derived on the basis of data available during 2011–2018 (COPD prevalence = 5.1 [3.9–6.3] in 2018).

## Discussion

An estimated 14.2 million (6.5%) U.S. adults had physician-diagnosed COPD in 2021. Overall prevalence remained unchanged since 2011. These results are consistent with overall COPD mortality rates, which remained unchanged during 1999−2019 (*4*). The prevalence of COPD among adults aged <45 years declined from 2011 to 2021, consistent with the trend during 1999–2011 (*3*). One reason might be the more pronounced decline in prevalence of current smoking among adults aged 18–44 years (36.4% relative decline) than among those aged 45–64 years (22.6%) and those aged ≥65 years (2.1%) from 2005 to 2015 (*5*); cigarette smoking is the dominant cause of COPD among U.S. adults.^†††^ Explanations for the higher prevalence in COPD among those living in micropolitan and noncore counties might include the persistently high prevalence of smoking among adults in rural areas (*6*), the lower rates of persons quitting smoking (*7*), and the increasing proportion of older adults living in rural areas.^§§§^ The variation in the prevalence of COPD by states is likely related to factors including differences in smoking rates, occupations or industries with higher risk for COPD, and access to health care for screening and detection of COPD (*8*,*9*).

Approximately 25% of adults with COPD (3.8 million) reported having never smoked, similar to 1988–1994 (*10*). In addition to cigarette smoking, secondhand smoke and occupational and environmental exposures are also risk factors for developing COPD among nonsmokers (*8*). Therefore, promotion of smoke-free environments^¶¶¶^ and workplace interventions (e.g., raising awareness of harmful work-related respiratory exposures, elimination or substitution of hazardous exposures, and improving ventilation) can help reduce or eliminate COPD-related risk factors.****

### Limitations

The findings in this report are subject to at least four limitations. First, the diagnosis of COPD, sociodemographic characteristics, and smoking status are all self-reported, and might be subject to recall and social desirability bias. Second, potential systematic bias resulting from low response rates might affect the results. The flat overall trend is also observed in the 2014–2018 NHIS,^††††^ suggesting that nonresponse bias did not significantly affect the conclusions of this report. Third, because there were no differences in COPD prevalence in 2020 or 2021 relative to 2019, it appears unlikely that the COVID-19 pandemic influenced reporting of physician-diagnosed COPD. Finally, the findings might not be extrapolated to adults in long-term care facilities, or in prisons, or those without a telephone because BRFSS collects data only from noninstitutionalized adults with a landline or mobile telephone.

### Implications for Public Health Practice

The COPD National Action Plan provides a comprehensive framework for developing and implementing COPD prevention, treatment, and management strategies.^§§§§^Patient and population-based initiatives focusing on COPD prevention (e.g., smoking cessation, smoke-free policies, and workplace interventions), early-diagnosis, treatment (e.g., medication and oxygen therapy), and management (e.g., access to pulmonary rehabilitation and caregiving, efforts to prevent exacerbations) might reduce COPD prevalence, slow the progression of the disease, and lessen symptoms. Although smoking is one of the main risk factors for COPD, it is important that initiatives include strategies for the 25% of U.S. adults with COPD who reported having never smoked. Strategies can be tailored to address the prevention of COPD-related risk factors and the needs of adults disproportionately affected by COPD, including persons aged ≥75 years, those who ever smoked, and residents of rural areas. For example, residents of rural areas have less access to pulmonologists (*9*). Implementation of COPD programs designed for rural communities can address the challenges that people from these areas face, including higher prevalence of tobacco use, cultural barriers, poverty, and lack of specialists or transportation.^¶¶¶¶^

## Footnotes

*
https://www.cdc.gov/brfss/

†
https://www.cdc.gov/brfss/annual_data/2011/pdf/2011_Summary_Data_Quality_Report.pdf

§
https://www.cdc.gov/brfss/annual_data/2021/pdf/2021-DQR-508.pdf

¶ The sample of 386,439 respondents in 2021 does not include those in Florida. Florida was unable to collect data during enough months to meet the minimum requirements for inclusion in the 2021 public-use dataset.

**
https://www.cdc.gov/nchs/data/statnt/statnt20.pdf

†† As defined in the CDC National Center for Health Statistics 2013 Urban-Rural Classification Scheme for Counties with six urbanization levels: four metropolitan (large central metropolitan, large fringe metropolitan, medium metropolitan, and small metropolitan) and two nonmetropolitan (micropolitan and noncore). https://www.cdc.gov/nchs/data/series/sr_02/sr02_166.pdf

§§
https://surveillance.cancer.gov/joinpoint/

¶¶
https://www.cdc.gov/nchs/data/series/sr_02/sr02_179.pdf

*** 45 C.F.R. part 46.102(l)(2), 21 C.F.R. part 56; 42 U.S.C. Sect. 241(d); 5 U.S.C. Sect. 552a; 44 U.S.C. Sect. 3501 et seq.

†††
https://stacks.cdc.gov/view/cdc/21569

§§§
https://www.census.gov/content/dam/Census/library/publications/2019/acs/acs-41.pdf

¶¶¶
https://www.cdc.gov/tobacco/secondhand-smoke/index.html

†††† https://www.lung.org/research/trends-in-lung-disease/copd-trends-brief/copd-prevalence

§§§§
https://www.nhlbi.nih.gov/health-topics/education-and-awareness/COPD-national-action-plan

¶¶¶¶
https://www.ruralhealthinfo.org/toolkits/copd
